# Functional assessment in children undergoing extracorporeal membrane oxygenation using the functional status scale: a scoping review

**DOI:** 10.3389/fped.2026.1910668

**Published:** 2026-09-23

**Authors:** Jhon Jairo Botello Jaimes, Luisa Fernanda Cornejo Castaño, Luis Fernando Sánchez Estrada, Angie Katherine Turriago Castañeda

**Affiliations:** 1 Facultad de Ciencias de la Salud, Corporación Universitaria Remington, Medellin, Colombia; 2 Facultad de Ciencias de la Salud, Universidad ICESI, Cali, Colombia

**Keywords:** adolescent, children, extracorporeal membrane oxygenation, functional status scale, infant, neonate, pediatric intensive care

## Abstract

As interest in the long-term outcomes of Extracorporeal Membrane Oxygenation (ECMO) grows, the Functional Status Scale (FSS) has emerged as an important tool for characterising rehabilitation needs in this patient population. This scoping review aimed to synthesise the functional outcomes assessed using the FSS, describe changes in these outcomes from baseline through discharge and during follow-up, examine the methodologies used to assign and interpret scores, and identify the main limitations and gaps in knowledge. The review was conducted according to the JBI methodology and the PRISMA-ScR guidelines, with the protocol registered in OSF (DOI: 10.17605/OSF.IO/AW287). We searched MEDLINE/PubMed, Ovid Emcare, and Scopus for studies of patients aged 0–18 years who underwent ECMO. We included 25 publications, predominantly retrospective cohorts from U.S. centres, with heterogeneous populations, indications, and ECMO modalities. The FSS primarily assessed functional status at discharge and new morbidity compared with baseline. Survivors generally exhibited mild to moderate functional impairment, primarily in feeding and respiratory function. Eight studies included measurements taken after discharge or discontinuation of support and described improvement in some survivors; however, a normal FSS score could coexist with neurobehavioral abnormalities the scale did not detect. No study formally evaluated the metric properties of the FSS specifically in this population. Prospective studies with standardised procedures, longitudinal measurements, and complementary assessments of neurodevelopment, adaptive behaviour, and quality of life are needed. Furthermore, qualitative studies could broaden our understanding of the post-ECMO experience by exploring the perspectives of patients and their families, changes in family dynamics, care and rehabilitation needs, and the influence of the social, educational, and community environment on the recovery process.

**Systematic Review Registration:**

https://osf.io/aw287/overview.

## 1 Introduction

Extracorporeal membrane oxygenation (ECMO) is a life-support technology that provides circulatory and pulmonary support to pediatric patients with severe cardiac and respiratory failure (1). It has been reported that more than 30,000 pediatric patients underwent this procedure between 1990 and 2020, with survival rates of approximately 61% for respiratory ECMO and 54% for cardiac ECMO, according to the Extracorporeal Life Support Organisation (ELSO) registry (2, 3). Given the potential for significant benefits and improved survival among critically ill pediatric and neonatal patients undergoing this procedure, it is essential to assess functional status and long-term morbidity following ECMO treatment (4, 5). Consequently, the current discussion goes beyond the effectiveness of the therapy in terms of survival, raising the need for a comprehensive assessment of the quality of life and functional status achieved following extracorporeal support (6). Research indicates that a substantial proportion of ECMO survivors may experience new health problems that affect their long-term quality of life, making the assessment of their functional status not only relevant but essential for informing rehabilitation strategies and resource allocation (2, 6).

The Functional Status Scale (FSS), developed and validated by Pollack et al. (7), assesses the functional status of critically ill pediatric patients treated in intensive care units. The scale comprises six domains: mental status, sensory function, communication, motor function, feeding, and respiratory status. It was developed in response to the need for a quantitative, rapid, reliable, and clearly defined tool with minimal subjectivity. Furthermore, it was designed to be applied across a wide age range and at different stages of clinical care (7). Its practicality facilitates its use both in clinical assessment and in studies involving large samples. The authors state that it was not intended to predict long-term outcomes (7). This same research group defined a cutoff point for new morbidity as a score that was 3 points or more higher than the baseline score (8). This threshold was established by expert consensus, as it was considered a clinically relevant functional change and corresponded approximately to the difference observed in the FSS between the “normal function” and “moderate disability” categories of the Pediatric Overall Performance Categories (POPC) (8).

Given its practicality, the scale has been widely adopted by researchers interested in measuring the functional outcomes of pediatric patients undergoing ECMO (9–11). However, although the scale has been extensively validated in general pediatric intensive care unit populations, questions remain regarding its application in patients undergoing ECMO, particularly due to the clinical heterogeneity of this population and the variability in the contexts and methods in which the scale has been applied (5, 12).

Therefore, this scoping review aimed to map and synthesize the available evidence on the use of the FSS in pediatric patients receiving ECMO support. Specifically, we sought to characterize the clinical and research contexts in which the FSS has been applied, the outcomes assessed, the timing of measurements, and its utility for evaluating and monitoring functional status. We also aimed to identify knowledge gaps and opportunities to standardize its application in this population. To the best of our knowledge, no previous evidence synthesis has specifically integrated these aspects in a pediatric population undergoing ECMO.

## 2 Methods

The scoping review was conducted following the JBI methodology for scoping reviews and the PRISMA-ScR extension for scoping reviews (13). The protocol for this review has been registered on the Open Science Framework (OSF) for public access via the following link: DOI 10.17605/OSF.IO/AW287.

### 2.1 Inclusion criteria

Studies were included that involved patients from birth through age 18 who were hospitalised in pediatric and/or neonatal intensive care units, with no restrictions based on diagnosis or clinical condition. The review considered studies that described the application or clinical use of the Functional Status Scale (FSS) in this context. Studies were eligible if they were conducted in settings where patients required ECMO therapy during their intensive care stay. Original primary studies and those based on secondary data sources were considered, including analytical observational designs such as prospective and retrospective cohorts, single-centre or multicenter studies, case-control studies, and analytical cross-sectional studies, as well as methodological studies evaluating the measurement properties of the FSS, such as its validity, reliability, and sensitivity to change. Grey literature was not included, as this review focused on evidence published in peer-reviewed scientific journals. Publications in English and Spanish were included with no time restrictions. Opinion pieces, letters to the editor, and texts not based on empirical evidence were excluded.

### 2.2 Search methods

A systematic search (see Supplementary Table 1) was conducted in MEDLINE (PubMed), Emcare (Ovid), and Scopus between September 2025 and February 2026, and was updated through a second search in August 2026. To this end, combinations of keywords and MeSH terms were used, linked by using Boolean operators and truncation. Following the JBI methodology, the search was conducted in three steps. First, an initial limited search was performed in PubMed to identify relevant articles on the topic. Next, we used the words in the titles and abstracts of the relevant studies, as well as the terms used for indexing, to construct a comprehensive search strategy. Finally, we adapted this strategy, including all identified keywords and index terms, to the specific syntax of each database. In addition, the reference lists of all included evidence sources were reviewed to identify additional potentially eligible studies.

### 2.3 Selection of studies

All identified citations were compiled and uploaded into the Rayyan bibliographic software (14), where duplicates were removed. Following a pilot study, two independent reviewers assessed the titles and abstracts to determine whether they met the review's inclusion criteria. Potentially relevant sources were retrieved in full, and their citation details were imported into the Mendeley reference manager (V2.149.0) for management, evaluation, and review. Two additional reviewers evaluated the full text of the selected citations in detail against the inclusion criteria. The PRISMA flow diagram (Figure 1) shows the reasons for excluding evidence sources after full-text review. Reviewers resolved disagreements through discussion and consensus.

**Figure 1:**
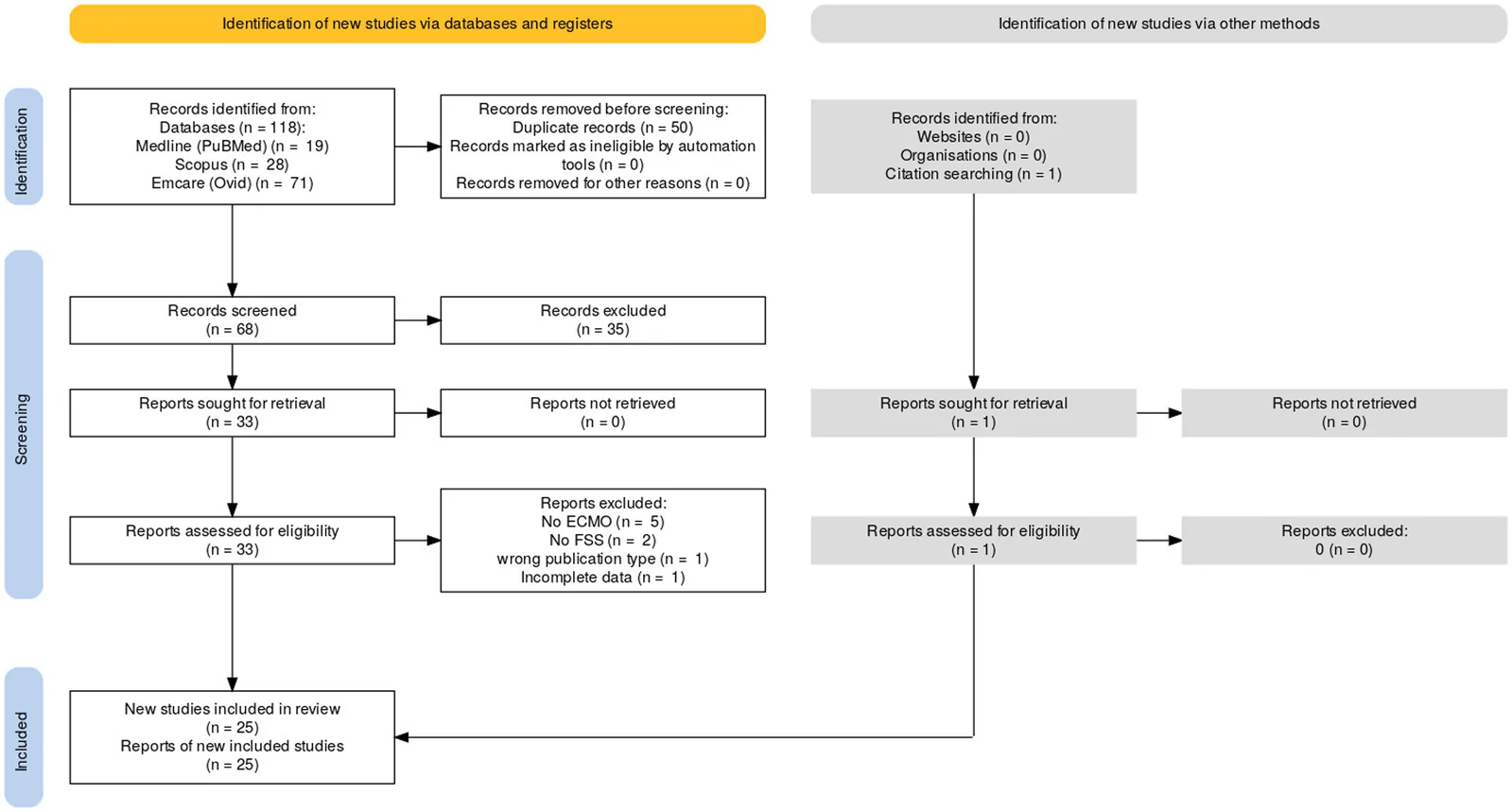
PRISMA flowchart of the studies included in the analysis.

### 2.4 Data extraction and analysis

Data extraction was performed using a form specifically designed by the reviewers for this scoping review. Prior to the final data extraction, a pilot test was conducted to evaluate the clarity, relevance, and applicability of the included variables and to make any necessary adjustments. Three reviewers then independently extracted data from the included studies. During this process, as relevant elements not initially considered were identified, the data extraction tool was modified to incorporate additional variables necessary for a more complete characterisation of the evidence. All reviewers discussed and approved these modifications by consensus before incorporating them into the tool. Likewise, reviewers discussed and resolved discrepancies identified during data extraction by consensus.

For each study, information was extracted regarding its general characteristics (author, year of publication, country, and study design), population characteristics (age, clinical condition, and indication for ECMO), and aspects related to the clinical context and extracorporeal support (ECMO modality, cannulation site, duration of therapy, and major reported complications). Regarding the concept of interest, information was collected on the application of the Functional Status Scale (FSS), including its purpose, measurement time points, reported functional outcomes, and associated outcomes. Additionally, the operational definitions used in each study, such as new morbidity or functional deterioration, were extracted.

For the analysis and synthesis of the findings, a descriptive qualitative analysis with a deductive approach was conducted, organised into three previously defined conceptual domains: characteristics of the population and ECMO support; use and measurement methodology of the FSS, including an evaluation of its measurement properties when this information was available; and, finally, functional outcomes and post-ECMO trajectory. The findings corresponding to these domains were integrated through a narrative synthesis of the included literature.

## 3 Results

### 3.1 Search and selection of studies

A total of 118 references were identified from the three databases consulted. After removing duplicates, 68 articles were screened. Next, 35 references were excluded after reviewing titles and abstracts. Thirty-three articles were read in full, of which 24 met all the established criteria. Figure 1 shows the reference selection flowchart. Reasons for excluding references included an incorrect publication type (letter to the editor), 5 studies in which the study population did not undergo ECMO, 2 studies that did not assess functional status using the FSS, and 1 reference that, being a conference abstract, did not provide sufficient data for inclusion in the analysis. One additional article was included following a review of the references cited in the included articles. Ultimately, 25 references were included in the analysis.

### 3.2 Included studies

The included studies were predominantly observational, with most using retrospective cohort designs (Table 1). Of the 25 publications analysed, 16 were studies involving retrospective data collection (10, 11, 15–27) and a case series (28); four were secondary analyses of a multicenter database with prospective data collection (Collaborative Pediatric Critical Care Research Network, BATE database) (9, 29, 30, 35); three had a cross-sectional design (30–32); and one was a prospective observational cohort study with long-term follow-up (33). Additionally, two studies were identified whose primary objective was medium- and long-term functional follow-up at time points ranging from three months to one year (21, 34). Three conference proceedings were included (19, 22, 23).

**Table 1:** Characteristics of the included studies.

Author, year	Country	Design	*n*
Cashen et al. 2017 (9)	USA	Secondary analysis (CPCCRN/BATE)	514 (282 survivors)
Beshish et al. 2018 (11) (ECPR)	USA	Single-center retrospective cohort	80 (38 survivors)
Cashen et al. 2018 (30) (hyperoxia/hypocapnia)	USA	Secondary analysis (CPCCRN/BATE)	484 (269 survivors)
Cashen et al. 2018 (29) (therapeutic hypothermia)	USA	Secondary analysis (CPCCRN/BATE)	267 (154 survivors)
Sirignano et al. 2018 (19)	USA	Single-center retrospective cohort	21 survivors
Ruth et al. 2022 (28) (septic shock)	USA	Retrospective case series	14 (8 survivors)
Shukla et al. 2021 (35)	USA	Secondary analysis (CPCCRN/BATE)	421 (236 survivors)
Sperotto et al. 2021 (20)	USA	Single-center retrospective cohort	182 (88 survivors)
Yuhang et al. 2022 (15)	China	Single-center retrospective cohort	27 (18 survivors)
Beshish et al. 2023 (36) (multicenter)	USA	Multicenter retrospective cohort	1,325 runs (746 survivors)
Beshish et al. 2024 (10) (Norwood, short-term)	USA	Single-center retrospective cohort	65 (27 survivors)
Beshish et al., 2024 (18) (Norwood, hyperoxia)	USA	Single-center retrospective cohort	65 (27 survivors)
Mattke et al. 2025 (34)	Australia	Cross-sectional survey	20 (12 survivors)
McCabe et al. 2024 (32)	USA	Prospective cross-sectional	180 patient encounters (37 attending intensivists)
Molloy et al. 2024 (21)	USA	Single-center retrospective cohort	42 survivors
Turner et al. 2024 (33)	USA	Prospective cohort of survivors	41 survivors
Beshish et al. 2025 (17) (VV-ECLS)	USA	Single-center retrospective cohort	110 (81 survivors)
Beshish et al. 2025 (16) (VA-ECLS)	USA	Single-center retrospective cohort	229 (127 survivors assessed)
Piñeres et al. 2025 (23) (awake ECMO)	Colombia	Single-center retrospective cohort	155 (42 extubated on ECMO)
Piñeres et al. 2025 (22) (functional outcomes)	Colombia	Single-center retrospective cohort	59
McGetrick et al. 2026 (24)	USA	Single-center retrospective cohort	75 (49 survivors)
Rabinowitz et al. 2026 (25)	USA	Single-center retrospective cohort	47 infants (21 survivors)
Ruth et al. 2026 (26) (propensity-matched)	USA	1:1 propensity-score-matched retrospective cohort	81 ECMO survivors and 81 non-ECMO controls
Saini et al. 2026 (27)	USA	Single-center retrospective cohort	229 (126 survivors assessed)
Sethi et al. 2026 (31)	USA	Prospective cross-sectional (planned secondary analysis)	80 surveys (21 fellows)

CPCCRN/BATE, Collaborative Pediatric Critical Care Research Network, Bleeding and Thrombosis during ECMO database; ECPR, extracorporeal cardiopulmonary resuscitation; VA/VV, venoarterial/venovenous.

The sample size ranged from 14 (28) to 746 patients (36), including both neonatal and pediatric populations, for a cumulative total of approximately 4,900 patient records or clinical visits, although in some cases there was overlap in data sources across several studies (9, 29, 30, 35). Some studies reported low median ages, reflecting a predominance of infants or neonates (10, 16, 17, 22, 23, 27, 36), while others included more heterogeneous cohorts with age ranges extending into late adolescence (33). Studies with post-discharge evaluations included follow-up at three, six, nine, and twelve months, as well as evaluations conducted several years after ECMO (33). The longest follow-up periods were 5.5 years in Turner et al. (33). and 6.3 years in Mattke et al. (34); in the former, the participants' age at follow-up reached and exceeded 10 years. Most of the studies were conducted at high-complexity centres in the United States (19 out of 23), with two studies from Latin America (22, 23), one from Asia (15), and one from Oceania (34).

Several included publications share a lead author or data source. However, they were retained as separate records because they addressed different research questions. Four publications are secondary analyses of the same multicenter cohort from the Collaborative Pediatric Critical Care Research Network [CPCCRN, “Bleeding and Thrombosis During ECMO” (BATE) project database] (9, 29, 30, 35), with different objectives and subsamples. Six additional publications in which Beshish et al. are listed as first authors were also included, corresponding to different research questions (10, 11, 16–18, 36). Among these, the two publications from 2024 share the same cohort of 65 neonates who underwent Norwood surgery (10, 18). One aimed to describe short-term outcomes and risk factors, using FSS as a descriptive measure (10), while the other evaluated the association between hyperoxia and mortality, with FSS as a secondary outcome and blinded evaluators (18). The two papers by Piñeres et al. come from the same Colombian center and describe different populations and objectives (22, 23).

Notably, the only study comparing a cohort of patients who underwent ECMO with a non-ECMO control group found that, although ECMO patients had greater functional impairment at discharge (median FSS 10 vs. 8), this difference was not statistically significant (*p* = 0.4). Furthermore, after adjusting for disease severity (PRISM-3) and hours of mechanical ventilation, the use of ECMO was not significantly associated with changes in the FSS (*p* = 0.205) (26).

The studies by McCabe et al. (32) and Sethi et al. (31) did not strictly meet the inclusion criteria, since their study population had not received ECMO support at the time the scale was administered; however, it was decided to include them because they document a different application of the FSS not described in the rest of the evidence, since the scale was not used as a functional outcome but rather as an exposure variable to classify patients as candidates or non-candidates for ECMO therapy.

### 3.3 Characteristics of the population and ECMO support

Participants' ages spanned a wide range, from newborns intubated during the first hours or days of life to adolescents (Table 2). The cohorts with the highest proportion of neonates or very young infants included patients with complex congenital heart defects, univentricular physiology following Norwood surgery, congenital diaphragmatic hernia, meconium aspiration syndrome, persistent pulmonary hypertension, and refractory cardiopulmonary failure (10, 16, 17, 27, 29, 30, 34, 35). In these studies, median ages ranged from 18.1 h of life in Mattke et al. (34) and 6 days in the post-Norwood cohorts (10, 18), to 2.5–4.9 months in studies by Beshish et al. (16, 17, 36). Rabinowitz et al. (25) included infants under one year of age exclusively, with a median age of 16 days. Other cohorts featured older ages or more heterogeneous distributions and consisted primarily of patients with fulminant myocarditis, septic shock, viral illnesses, and acute respiratory failure (15, 24, 26, 28, 31, 32). The median or mean ages ranged from 2.4 to 6.2 years, although several studies included participants from the neonatal period through adolescence. Patients who received extracorporeal cardiopulmonary resuscitation were also included (11, 20).

**Table 2:** Characteristics of the population and ECMO support.

Author, year	Age	Main admission diagnosis	ECMO indication	Mode and cannulation	Duration (days)	Main complications
Cashen et al. 2017 (9)	< 19 years; 52% neonates	Respiratory 46%	Respiratory 46%, cardiac 40%, ECPR 14%	VA 84%	5 (3–9)	Hemorrhage 70%, neurologic 37%–42%
Beshish et al. 2018 (11)	Median 40 days	Congenital or acquired heart disease	ECPR 100%	VA 100%; central 55%	3.7–4	Radiologic CNS injury in 74% of those with new morbidity
Cashen et al. 2018 (30)	< 19 years	Congenital heart disease 36%	Respiratory 47%, cardiac 42%, ECPR 11%	VA 87%	5.2 (2.8–9.5)	Hemorrhage 71%, neurologic 40%
Cashen et al. 2018 (29)	Neonates < 30 days; median 3.0 days	Respiratory failure/persistent pulmonary hypertension	Respiratory 57%, cardiac 34%, ECPR 9%	VA 87%	5.3 control vs. 6.7 hypothermia	Hemorrhagic 58%–75%; intracranial hemorrhage 47/267 (18%)
Sirignano et al. 2018 (19)	> 30 days to 18 years	Pediatric non-cardiac	Predominantly respiratory	VV 71%	9	NR
Ruth et al. 2022 (28)	Median 104.5 months	Refractory septic shock	Septic shock 100%	VA 100%; central in 9/14	6.1	Hemorrhagic 10/14
Shukla et al. 2021 (35)	< 18 years; 53% neonates	Respiratory 50%, cardiac 34%	Respiratory 54%, cardiac 31%, ECPR 15%	VA 83%/VV 17%	5 (3–9)	Not reported separately
Sperotto et al. 2021 (20)	Median 148 days	Congenital heart disease	ECPR 100%	VA 100%	4	Hypoxic-ischemic injury 47%, hemorrhage 41%
Yuhang et al. 2022 (15)	Median 36 months (21–114)	Fulminant myocarditis (13/27)	Myocarditis, ARDS and septic shock	VA 12/VV 11/ECPR 4; peripheral and central	4	Lower-limb deep vein thrombosis 50%
Beshish et al. 2023 (36)	Median 3.1 months (0–18 years)	Respiratory failure	Respiratory 47%	VA 51%/VV 49%; peripheral 76%	5.5 (3.5–8.3)	Cardiovascular 59%
Beshish et al. 2024 (10)	Neonates; median 6 days	Hypoplastic left heart syndrome 77%	Postoperative cardiac	VA; site NR	3.7	Renal
Beshish et al., 2024 (18)	Neonates; median 6 days	Single-ventricle physiology/hypoplastic left heart 77%	Postoperative cardiac 59%, ECPR 41%	VA 100%; central 67%	3.4 without vs. 5.8 with hyperoxia (*p* = 0.005)	Cardiovascular 88%, renal 55%
Mattke et al. 2025 (34)	Cannulation at 18.1 h of life	Congenital diaphragmatic hernia	Respiratory	VA 13/VV 5/2 conversions	13.6 (5.8–24.3)	Hemorrhagic (cause of death)
McCabe et al. 2024 (32)	Median 3.0 years (1.3–9.0)	Acute respiratory failure	Theoretical candidacy (patients not cannulated)	Not applicable	Not applicable	Not applicable
Molloy et al. 2024 (21)	Median 1.7 years	Cardiac 50%	Cardiac 50%	VA 93%	3.9 (2.3–5.9)	Neurologic 20% (seizures or ischemia)
Turner et al. 2024 (33)	Median 2.4 years at admission	Viral illness	Cardiac 59%, ECPR 27%	VA 78%; peripheral 78%	6	Intracranial hemorrhage 38%
Beshish et al. 2025 (17)	Median 4.9 months; 57% neonates	ARDS, meconium aspiration, persistent pulmonary hypertension	Pulmonary 86%, cardiac 7%, ECPR 7%	VV 100%	5.8 (4.1–12.0)	Renal 68%, mechanical 58%, cardiovascular 41%
Beshish et al. 2025 (16)	Median 2.5 months; 73% neonates	Refractory cardiopulmonary failure	Cardiac 49%, ECPR 28%, pulmonary 23%	VA 100%	4.6 (2.7–7.7)	Renal 54%, cardiovascular 46%, hemorrhagic 45%
Piñeres et al. 2025 (23)	Median 9 months	Acute respiratory distress syndrome	Respiratory	VA; peripheral	8 (IQR 6)	CNS hemorrhage
Piñeres et al. 2025 (22)	Median 3.0 months (0.4–42)	Pediatric non-cardiac	Not specified	NR	NR	ECMO-related complications in 55.9%
McGetrick et al. 2026 (24)	Median 24 months; 31% neonates	Refractory cardiopulmonary failure	Cardiac 56%, respiratory 44%	VA 80%/VV 20%	5.1 (3.8–12.0)	Acute neurologic injury; renal replacement therapy 37%
Rabinowitz et al. 2026 (25)	Infants < 1 year; median 16 days	Congenital heart disease 62%	Cardiac 62%, respiratory 21%, pulmonary hypertension 17%; ECPR 34%	VA 92%/VV 9%; peripheral 64%	5.6 (2.7–10.9)	Abnormal head ultrasound 70%; abnormal EEG 32%
Ruth et al. 2026 (26)	Mean 6.2 years (cases) and 6.1 years (controls)	Respiratory, followed by infectious	Non-cardiac respiratory or infectious failure	VV 73%/VA 27%; right internal jugular	8.8 (VA 8.1 vs. VV 10.1)	Neurologic: 3 hemorrhagic and 2 ischemic
Saini et al. 2026 (27)	Median 2.5 months; 73% neonates	Congenital heart disease or other causes of cardiac failure	Cardiac 49%, ECPR 28%, pulmonary 23%	VA 100%	4.6 (2.7–7.7)	Renal 54%, cardiovascular 46%, hemorrhagic 45%
Sethi et al. 2026 (31)	Median 3.5 years (1.1–9.0)	Acute respiratory failure	Theoretical candidacy (patients not cannulated)	Not applicable	Not applicable	Not applicable

ECPR, extracorporeal cardiopulmonary resuscitation; NR, not reported; ARDS, acute respiratory distress syndrome; CNS, central nervous system; VA/VV, venoarterial/venovenous.

Respiratory indications were the most common in several of the larger cohorts, 47% in Beshish et al. (36), 46% in Cashen et al. (9), 47.2% in Cashen et al. (30), 54% in Shukla et al. (35), and 85.5% in Beshish et al. (16), whereas the cardiac indication predominated in Saini et al. (27) at 48.9%, in Turner et al. (33) at 59%, and in McGetrick et al. (24) at 56%. In Rabinowitz et al. (25), 62% of patients received ECMO for a cardiac indication. ECPR was the sole indication in Beshish et al. (11) and Sperotto et al. (20), and accounted for intermediate proportions in Beshish et al. (10), Saini et al. (27), Shukla et al. (35), and Cashen et al. (29).

The use of venoarterial ECMO was predominant, with rates of 84% in Cashen et al. (9), 86.7% in Cashen et al. (30), 86.5% in Cashen et al. (hypothermia) (29), 83% in Shukla et al. (35), 80% in McGetrick et al. (24), 78% in Turner et al. (33), and 93% in Molloy et al. (21); all-venoarterial cohorts were reported in Saini et al. (27), Sperotto et al. (20), and Beshish et al. (Norwood) (10). Two studies had a predominance of venovenous access: Sirignano et al. (19) at 71.4% and Beshish et al. (17) with a 100% venovenous cohort. Beshish et al. (36) was the only study with a balanced distribution between the two modalities (51% venoarterial versus 49% venovenous). In the comparative cohort by Ruth et al. (26), 72.8% received venovenous support and 27.2% received venoarterial support.

The cannulation site was predominantly peripheral in the mixed cohorts, at 76% in Beshish et al. (36) and 78% in Turner et al. (33) and central in the cardiac and ECPR cohorts, at 55% in Beshish et al. (11), 67% in Beshish et al. (Norwood) (10), and in the entire series by Ruth et al. (28), which focused on central venoarterial cannulation for refractory septic shock. In the comparative study by Ruth et al. (26), cannulation was performed via the right internal jugular vein.

Regarding the duration of therapy, most studies reported medians ranging from 3.4 to 5.8 days (9, 15–18, 20, 23–25, 27, 35, 36). Molloy et al. (21) reported 94.6 h (IQR 56–142). The longest durations were reported by Sirignano et al. (19), at 9 days; Ruth et al. (26), at 8.8 days; and Mattke et al. (34), at 13.6 days in survivors of congenital diaphragmatic hernia.

Renal complications were reported in 68% of the venovenous cohort in Beshish et al. (17), in 55% of post-Norwood patients, and in 54% of the venoarterial cohorts in Beshish et al. (27) and Saini et al. (27). Cardiovascular events were reported in 59% of the cases in Beshish et al. (36), in approximately 88% of the post-Norwood study on hyperoxia, and in 46% of the VA-ECLS cohorts. Among neurological complications, intracranial hemorrhage was described in 38% of cases in Turner et al. (33), radiological lesions of the central nervous system in 74% of patients with new morbidity in Beshish et al. (11), abnormalities on cranial ultrasound in 70% of patients in Rabinowitz et al. (25), and acute neurological damage in McGetrick et al. (24). In the latter study, neurological causes accounted for 34.6% of deaths.

### 3.4 Use and measurement methodology of the FSS

The FSS was used for various purposes in the 25 included studies (Table 3). In 12 publications, it was primarily used to describe functional status, its progression, or the onset of new morbidity as an outcome of interest (9–11, 15, 19, 21–23, 25, 26, 33, 34, 36). Nine studies incorporated it as a secondary outcome in research focused on other exposures or prognostic tools, including hyperoxia, hypocapnia, therapeutic hypothermia, vasoactive scores, oxygen delivery markers, and automated pupillometry (16–18, 24, 25, 27, 30, 35). One study included it as a component of a primary composite outcome within a predictive model (20); two used it as an exposure variable for classifying ECMO eligibility (31, 32); and one used it to capture morbidity in a case series (28). Its most frequent use, therefore, was assessing functional status as a clinical outcome, particularly at hospital discharge.

**Table 3:** Measurement methodology of the FSS, survival and functional outcomes.

Author, year	Role of the FSS	Assessment time points and follow-up	Source of the score	Baseline → discharge FSS	Survival to discharge	New morbidity (*Δ* ≥ 3)	Unfavorable outcome (*Δ* ≥ 5)	Most affected domain
Cashen et al. 2017 (9)	Primary outcome	Discharge only	Prospective registry (BATE database)	Not measured → 32% good, 40% mildly, 24% moderately, 5% severely abnormal	55% (282/514)	Not assessed (no baseline FSS)	Not assessed	NR
Beshish et al. 2018 (11)	Global measure of function	Admission and discharge	Retrospective chart review	6 (6–6) → 9 (8–11)	48% (38/80)	50% (19/38)	32% (12/38)	Feeding
Cashen et al. 2018 (30)	Secondary outcome	Discharge only	Prospective registry (BATE database)	Not measured → categorized	56% (269/484)	Not assessed (no baseline FSS)	Not assessed	NR
Cashen et al. 2018 (29)	Secondary outcome	Discharge only	Prospective registry (BATE database)	Not measured → categorized	58% (154/267)	Not assessed (no baseline FSS)	Not assessed	NR
Sirignano et al. 2018 (19)	Functional trajectory	Baseline, hospital discharge and rehabilitation discharge	Two independent reviewers (kappa 0.88–0.92)	Categorized	21 survivors, no in-hospital deaths	85.7% with worse FSS than baseline (mean delta 4.84)	NR	NR
Ruth et al. 2022 (28)	Morbidity in a case series	Baseline and discharge	Retrospective chart review	6.9 → 10 (means)	57% (8/14)	Not reported; all survivors increased their FSS	NR	NR
Shukla et al. 2021 (35)	Secondary outcome	Discharge only	Prospective registry (BATE database)	Not measured → 8 (7–10)	56% (236/421)	Not assessed (no baseline FSS)	2.5% (6/236) with FSS > 18	NR
Sperotto et al. 2021 (20)	Component of the composite outcome (death or FSS ≥ 16)	Admission, discharge and 6 months	Retrospective chart review	7 (6–10) → 8 (6–8)	48% (88/182); 43% at 6 months	Not reported (median delta 0)	1% of survivors with FSS ≥ 16	NR
Yuhang et al. 2022 (15)	Morbidity and overall prognosis	Admission, discharge and 1 year	Retrospective chart review	17 (14–20) → 30 (7–30)	67% (18/27); 44% at 1 year	33% (6/18)	NR	Motor and communication
Beshish et al. 2023 (36)	Primary outcome	Admission and discharge	Retrospective chart review	6.51 (SD 1.72) → 9.1 (SD 2.72)	56% (746/1,325)	40% (299/746)	16% (123/746)	Feeding
Beshish et al. 2024 (10)	Descriptive outcome	Admission and discharge	Retrospective chart review	6 → 8.48 (SD 1.25)	41.5% (27/65)	26% (7/27)	7% (2/27)	Feeding
Beshish et al., 2024 (18)	Secondary outcome	Baseline and discharge	Blinded retrospective chart review	6.0 ± 0 → 8.4 ± 1.2 without and 8.8 ± 1.4 with hyperoxia	41.5% (27/65)	26% (7/27); 21% vs. 38%	7% (2/27); 5% vs. 13%	Feeding
Mattke et al. 2025 (34)	Long-term function	Single assessment at a median of 6.3 years	Structured telephone interview with caregivers	No baseline → 6 (IQR 6–7) at follow-up	60% (12/20)	Not assessable (no baseline); 8/12 with normal function	—	Respiratory and feeding
McCabe et al. 2024 (32)	Exposure variable (ECMO candidacy)	PICU admission	Real-time prospective assessment	9.5 (6.0–15.0) at admission	Not applicable	Not applicable	Not applicable	Communication
Molloy et al. 2024 (21)	Functional status and trajectory	Admission, discharge, 9 and 12 months	Retrospective chart review	6 (6–6) → 8.0 (6.3–8.8)	Survivors only	24% (10/42)	NR	Respiratory and feeding
Turner et al. 2024 (33)	Functional status and trajectory	Baseline, discharge and follow-up at a median of 5.5 years	Prospective caregiver interview plus chart review	NR → 8 (6–9); 6 (6–7) at follow-up	Survivors only	28% (11/40)	NR	Feeding 40%
Beshish et al. 2025 (17)	Secondary outcome	Baseline and discharge	Blinded retrospective chart review	Not reported as a continuous value	74% (81/110)	27% (22/81)	7% (6/81)	NR
Beshish et al. 2025 (16)	Secondary outcome	Baseline and discharge	Retrospective scoring by the research team	Not reported as a continuous value	56% (mortality 44.5%)	24% (30/127); 22% vs. 29%	8% (10/127)	NR
Piñeres et al. 2025 (23)	New morbidity	Admission, discharge and 3 months	Not reported	6 (IQR 0) → 7 (IQR 1.5)	83% in extubated patients	9.5% at 3 months (new morbidity or unfavourable outcome)	—	NR
Piñeres et al. 2025 (22)	Functional outcome	Admission, discharge and 3 months	Not reported	NR	Not reported	15.3% (9/59)	3.4% (2/59)	NR
McGetrick et al. 2026 (24)	Secondary outcome (FSS ≥ 10)	Intensive care unit discharge	Assigned by the clinical care team	Not measured → 33/49 with FSS ≥ 10	65% (49/75)	Not assessed (no baseline FSS)	67% of survivors with FSS ≥ 10	NR
Rabinowitz et al. 2026 (25)	Secondary outcome and component of the composite (death or FSS ≥ 10)	Admission and discharge	Retrospective review of the electronic health record	10 (9–14; *n* = 33) → 9 (8–11; *n* = 21)	45% (21/47)	76% of survivors discharged with new technology dependence	68% (32/47) with a poor composite outcome	Feeding 75%
Ruth et al. 2026 (26)	Main outcome, compared with matched controls	PICU admission and discharge	Retrospective review of hospital databases	6 (6–7) → 10 (7–13); controls 6 (6–8) → 8 (6–12)	70% (81/116)	44% (36/81) vs. 30% (24/81) in controls	30% (24/81)	Feeding, followed by respiratory
Saini et al. 2026 (27)	Secondary outcome	Baseline and discharge	Blinded retrospective chart review	Not reported as a continuous value	55% (mortality 45%)	20.7–28.6% across hyperoxia severity groups (*p* = 0.88)	0%–9.3% (*p* = 0.81)	NR
Sethi et al. 2026 (31)	Exposure variable (ECMO candidacy)	PICU admission	Trained bedside nurses independent of the care team	7 (6–12) at admission	Not applicable	Not applicable	Not applicable	NR

SD, standard deviation; FSS, functional status scale; IQR, interquartile range; NR, not reported; PICU, pediatric intensive care unit.

With regard to measurement time points, 9 studies administered the scale at admission (or baseline) and at hospital discharge (9, 24, 29, 30, 35); 5 measured it only at discharge, without a baseline value for comparison (9, 24, 29, 30, 35); 2 measured it exclusively at admission, as part of the eligibility assessment (31, 32); and 6 studies (27.3%) included at least one measurement after hospital discharge. Sirignano et al. (19) added a measurement point not used by any other study, which was discharge from a pediatric inpatient rehabilitation unit. The follow-up periods were 3 months in Piñeres et al. (23), 6 months in Sperotto et al. (20), 9–12 months in Molloy et al. (21), 1 year in Yuhang et al. (15), a median of 5.5 years after decannulation in Turner et al. (33), and a median of 6.3 years after the event in Mattke et al. (34).

Most studies reconstructed both the premorbid baseline and the FSS at discharge through a retrospective review of medical records. Within this group, 3 studies specified that the scoring was performed by reviewers blinded to the exposure status (16, 18, 27). Analyses conducted using the CPCCRN (9, 29, 30, 35) employed prospective data collection by trained clinical research coordinators, who combined direct observation of the child, discussions with treating clinicians, and review of medical records. McCabe et al. (32) assessed the FSS in real time as part of a prospective cross-sectional survey, and Sethi et al. (31) relied on trained nursing staff independent of direct care, with the physicians surveyed blinded to the scores. Mattke et al. (34) conducted follow-up via structured telephone interviews with caregivers, and Turner et al. (33) via in-person interviews. In McGetrick et al. (24), scores were assigned by intensive care unit staff as part of institutional clinical practice. Only one of the 23 studies quantified inter-rater agreement, Sirignano et al. (19) reported, using weighted kappa, values of 0.877 for the baseline and 0.915 for the FSS at discharge (*p* < 0.0001).

Regarding the assignment of the baseline FSS in neonates intubated before reaching their premorbid functional status outside the hospital setting, Beshish et al. (10, 11, 16, 17, 36) and Saini et al. (27) assigned a normal baseline FSS of 6 to admissions occurring within the first 2 days of life, extending this convention to transfers from external institutions up to day 6 of life; Molloy et al. (21) assigned a baseline FSS of 6 to newborns transferred at 10 days of life or younger, and Turner et al. (33) applied a similar convention. The remaining studies included a substantial proportion of neonates. In Beshish et al. (Norwood) (10), the entire cohort was conventionally assigned a baseline score of 6.0 ± 0.

Operational definitions of the total score varied across studies. Six operationalizations were identified, an increase of 3 points or more from baseline as the definition of new morbidity; an increase of 5 points or more as an unfavorable functional outcome; an FSS score of 10 or higher as moderate or severe disability; an FSS score of 16 or higher as severe functional impairment; an FSS score of 18 or higher as an unfavorable outcome; and Pollack's original categories (6–7: good; 8–9: mildly abnormal; 10–15: moderately abnormal; 16–21: severely abnormal). Studies on ECMO candidacy also grouped baseline scores into categories of normal function or mild dysfunction and moderate or severe dysfunction (31, 32).

None of the included studies evaluated the measurement properties of the FSS in pediatric patients undergoing ECMO. No analyses of construct or criterion validity or sensitivity to change were identified in this population. The only metric indicator reported was the weighted kappa coefficient for interobserver agreement by Sirignano et al. (19).

Five studies used a second functional, neurobehavioral, or quality-of-life outcome measure. Turner et al. (33) quantified the relationship between the FSS and the Vineland Adaptive Behavior Scales, Third Edition, and found that higher composite scores on the Vineland-3 were associated with a lower FSS both at hospital discharge (R² = 0.12; *p* = 0.03) and at long-term follow-up (R² = 0.24; *p* = 0.002); this association remained after adjusting for age at catheterization, socioeconomic deprivation, and time to follow-up. Mattke et al. (34) administered the PedsQL alongside the FSS and described complementary domains, without calculating a formal correlation coefficient. Shukla et al. (35) used the Pediatric Cerebral Performance Category (PCPC) and Pediatric Overall Performance Category (POPC) at discharge alongside the FSS. Beshish et al. (36) compared new morbidity as defined by the FSS with structural neuroimaging findings, and Molloy et al. (21) documented a borderline association between seizures confirmed by electroencephalogram or cerebral ischemia and a greater increase in the FSS (*p* = 0.05).

### 3.5 Functional outcomes and post-ECMO course

Studies that reported baseline FSS scores generally documented scores close to the scale's minimum value (Table 3). A median of 6 (IQR 6–6) was reported in Beshish et al. (11) and Molloy et al. (21); a mean of 6.0 ± 0 in Beshish et al. (Norwood) (18); a score of 6 in Beshish et al. (short-term outcomes) (10); a median of 6 (IQR 0) in Piñeres et al. (23); a mean of 6.51 ± 1.72 in Beshish et al. (36); a mean of 6.9 in Ruth et al. (28); and a median of 7 (IQR 6–10) in Sperotto et al. (20). In the comparative study by Ruth et al. (26), the baseline median was 6 for both ECMO-treated survivors (IQR 6–7) and matched controls without ECMO (IQR 6–8). Rabinowitz et al. (25) reported a higher baseline median of 10 (IQR 9–14) in a cohort of infants with a high proportion of congenital heart defects.

The two studies on ECMO candidacy applied the FSS only at admission to patients who had not previously received this support. McCabe et al. (32) reported a median of 9.5 (IQR 6.0–15.0), while Sethi et al. (31) reported a median of 7 (IQR 6–12). In the latter study, 85% of patients had at least one chronic condition; the most common were chronic respiratory failure (31%), developmental delay (31%), and epilepsy or seizure disorders (23.5%). Seventy-five per cent were classified as having normal function or mild dysfunction, and 25% as having moderate or severe dysfunction, although another section of the same study reported proportions of 68.7% and 31.3%, respectively.

At hospital discharge, Beshish et al. (36) reported a mean FSS score of 9.1 ± 2.72; Beshish et al. (short-term outcomes) (10) 8.48 ± 1.25; and Beshish et al. (Norwood) (18) reported means of 8.4 ± 1.2 and 8.8 ± 1.4 in the groups without and with hyperoxia, respectively. The medians were 9 (IQR 8–11) in Beshish et al. (11), 8.0 (IQR 6.3–8.8) in Molloy et al. (21), 8 (IQR 7–10) in Shukla et al. (35), 8 (IQR 6–9) in Turner et al. (33), 10 (IQR 7–13) among ECMO survivors in Ruth et al. (26) and 8 (IQR 6–12) in their matched controls. Ruth et al. (28) reported a mean of 10, while Piñeres et al. (23) reported a median of 7 (IQR 1.5). In Rabinowitz et al. (25) the median among the 21 survivors was 9 (IQR 8–11). The studies by Beshish et al. (16, 17) and Saini et al. (27) did not present continuous baseline or discharge values, but rather proportions of new morbidity and adverse outcomes. Yuhang et al. (15) reported medians of 17 (IQR 14–20) at admission and 30 (IQR 7–30) at discharge.

Categorical distributions at discharge were available in the analyses derived from CPCCRN/BATE. In Cashen et al. (hypothermia) (29) the 140 survivors in the non-hypothermia group were distributed as follows: 32.9% with an FSS of 6–7, 44.3% with an FSS of 8–9, 19.3% with an FSS of 10–15, and 3.6% with an FSS of 16–21. Among the 14 survivors treated with hypothermia, these proportions were 35.7%, 57.1%, 7.1%, and 0%, respectively; the authors reported no statistically significant differences between groups. Cashen et al. (9) reported that, among survivors, 32% had good function, 40% had mild impairment, 24% had moderate impairment, and 5% had severe impairment. Shukla et al. (35) documented a median of 8 (IQR 7–10) and six of 236 survivors (2.5%) with an FSS > 18. McGetrick et al. (24) using an FSS cutoff of ≥ 10, reported that 33 of 49 survivors (67%) were discharged with moderate or severe disability; the remaining 16 survivors had an FSS below 10.

The proportion of new morbidity, defined as an increase of ≥ 3 points from baseline, varied across studies. It was reported in 50% of survivors in Beshish et al. (11), 40% in Beshish et al. (36), 33% in Yuhang et al. (15), 28% in Turner et al. (33), 27% in the venovenous cohort by Beshish et al. (17), 26% in the two post-Norwood publications by Beshish et al. (10, 18), 24% in Molloy et al. (21), 23.6% in the venoarterial cohort by Beshish et al. (16), and 15.3% in Piñeres et al. (22). Saini et al. (27) reported proportions ranging from 20.7% to 28.6% depending on the hyperoxia exposure category, with no statistically significant differences between the groups (*p* = 0.88).

In the comparative study by Ruth et al. (26) 44.4% of ECMO survivors showed an increase of ≥ 3 points, compared with 29.6% of the matched controls. Sirignano et al. (19) reported that 85.7% of participants had a worse FSS score at discharge than at baseline, with an average increase of 4.84 points. Ruth et al. (28) indicated that all survivors saw an increase in their scores, although they did not report the proportion corresponding to an increase of ≥ 3 points. Piñeres et al. (23) reported that 9.5% of patients experienced new morbidity or an unfavourable outcome at three months, without distinguishing between the two categories.

For an unfavourable outcome, defined as an increase of ≥ 5 points, the proportions were 32% in Beshish et al. (11), 30% among ECMO survivors in Ruth et al. (26), 16% in Beshish et al. (36), 8% in the venoarterial cohort by Beshish et al. (16), 7% in the two post-Norwood studies by Beshish et al. (10, 18), 7% in the venovenous cohort by Beshish et al. (17), and 3.4% in Piñeres et al. (22). Saini et al. (27) reported rates ranging from 0% to 9.3% depending on hyperoxia severity, with no significant differences between groups (*p* = 0.81). Beshish et al. (Norwood) (18) reported an unfavourable outcome occurred in 5% of survivors without hyperoxia and in 13% of those with hyperoxia, with no statistically significant difference.

Rabinowitz et al. (25) used a composite outcome defined as in-hospital death or an FSS score ≥ 10 at discharge. This outcome occurred in 32 of the 47 patients (68.1%). Among the 21 survivors, 16 (76.2%) were discharged with a new dependence on medical technology, including a nasogastric tube, gastrostomy or gastrojejunostomy, and tracheostomy.

The feeding domain was most frequently reported as affected. This finding was described by Beshish et al. (11), Beshish et al. (36), the two post-Norwood studies by Beshish et al. (10, 18), Turner et al. (33), Rabinowitz et al. (25), and Ruth et al. (26). Turner et al. (33) found that the feeding domain was impaired in 16 of 40 patients (40%). In Rabinowitz et al. (25), impairment was found in 75% of the patients evaluated. Molloy et al. (21) described impairment in the respiratory and feeding domains, while Ruth et al. (26) reported that the respiratory domain was the second most affected after feeding.

In the post-Norwood study on hyperoxia, the feeding domain increased from a baseline level of 1 to a mean of 2.9 ± 0.5 at discharge in the group without hyperoxia and to 3.0 ± 0 in the group with hyperoxia (18). Yuhang et al. (15) identified the motor and communication domains as the most affected. In the context of candidate assessment, McCabe et al. (32) identified the communication domain as the subscore associated with a lower probability that the patient would be considered a candidate for ECMO in the univariate analysis (OR = 0.44).

Eight studies included a measurement after hospital discharge or upon discontinuation of support. Piñeres et al. (23) and Piñeres et al. (22) conducted assessments at three months; in the second study, 84.7% of patients had normal function at that time. Sperotto et al. (20) assessed the FSS at six months as part of a composite outcome and reported a survival rate of 43%; 1% of survivors had an FSS ≥ 16. Molloy et al. (21) reported a median of 6.0 (IQR 6.0–8.0) at both nine and twelve months, compared with a median of 8.0 at discharge.

Yuhang et al. (15) evaluated survival and new morbidity at one year. Turner et al. (33) reported a median FSS of 6 (IQR 6–7) during follow-up at a median of 5.5 years after decannulation, compared with 8 (IQR 6–9) at discharge, and simultaneously applied the Vineland-3 scale to characterise neurobehavioral outcomes. Mattke et al. (34) conducted a single cross-sectional assessment at a median of 6.3 years after the event and reported a median FSS score of 6 (IQR 6–7); eight of the 12 survivors evaluated scored 6. Consequently, this result corresponds to long-term follow-up rather than a baseline measurement. Sirignano et al. (19) added an assessment upon discharge from inpatient pediatric rehabilitation and documented functional improvement in 84.6% of participants between admission and discharge from that unit.

Sperotto et al. (20) developed a predictive model that incorporated death or an FSS score ≥ 16 as a composite outcome following ECPR, with functional measurements taken at admission, discharge, and at six months. Other studies used the FSS as an outcome measure in relation to specific clinical predictors. Shukla et al. (35) evaluated the vasoactive-inotropic and vasoactive-ventilatory-renal scores prior to the initiation of ECMO; McGetrick et al. (24) examined the neurological pupillary index during the first 72 h; and Rabinowitz et al. (25) analysed markers of impaired oxygen delivery during the six hours prior to cannulation. Cashen et al. (9) identified renal failure and a longer length of stay in the intensive care unit as factors independently associated with a poorer functional status at discharge.

## 4 Discussion

This scoping review synthesised 25 publications that used FSS in neonatal and pediatric populations related to ECMO. The available evidence shows that the scale has been used primarily to describe the functional status of survivors at discharge and the onset of new morbidity, although it was also incorporated as a secondary outcome in prognostic studies, as a component of composite outcomes, during longitudinal follow-up, and, in two publications, as a variable associated with the assessment of eligibility for ECMO. Rather than showing a uniform functional pattern, the studies identify three general findings: functional impairment was common at discharge among survivors; it is primarily evident in feeding and respiratory function; and some of this impairment may improve during follow-up. However, the magnitude and progression of these changes cannot be estimated collectively because of clinical and methodological heterogeneity in the evidence.

In studies comparing baseline to discharge, median FSS scores typically increased by 2–3 points, from 6 to 8 or 9. Although these scores generally correspond to overall categories of mild dysfunction, they may indicate new needs for enteral feeding, respiratory support, medical devices, or rehabilitation. New morbidity, generally defined as an increase of ≥3 points, ranged from approximately 15% to 50% among studies that used this definition. Adverse outcomes, defined primarily as an increase of ≥5 points, were less frequent, although they reached rates close to 30% in some cohorts. This range reflects differences in age, diagnosis, indication and modality of ECMO, disease severity, survival, and the FSS scoring procedure, as well as variations in cutoff points.

The observed deterioration cannot be attributed exclusively to extracorporeal support. Patients undergoing ECMO have severe critical illness and are simultaneously exposed to mechanical ventilation, sedation, prolonged immobility, surgery, organ failure, and the risk of neurological injury, in addition to potential increased rehabilitation needs. The only study that compared ECMO survivors with propensity-score-matched PICU controls found a higher median FSS at discharge in the ECMO group 10 versus 8 and a higher proportion of new morbidity 44.4% versus 29.6%. However, the differences were not statistically significant, and ECMO use was not associated with changes in the FSS after adjusting for severity and duration of mechanical ventilation (26). This result does not demonstrate functional equivalence between the groups, but it does highlight the difficulty of distinguishing the effect of ECMO from that of the underlying disease and the course of hospitalisation; however, it should also be noted that the sample size of this study was small, which may have reduced its statistical power.

Feeding was the domain most frequently affected, followed by respiratory function. Consequently, a small increase in the total FSS score does not necessarily represent a minor clinical change: it may indicate the need for a nasogastric tube, gastrostomy, tracheostomy, supplemental oxygen, or other forms of support. The total score should therefore be interpreted alongside an analysis of the individual domains, as these allow more precise identification of post-discharge needs; however, several studies report only the overall FSS.

Longitudinal results indicate that functional status at discharge does not necessarily reflect the survivor's final condition, but the evidence does not allow for the identification of a single trajectory. Eight studies conducted some form of follow-up assessment after hospital discharge or discontinuation of ECMO support: two at three months, one at six months, one between nine and twelve months, one at one year, two after five years, and one upon discharge from a rehabilitation unit.

These results are consistent with functional recovery in a proportion of survivors, but they must be interpreted in light of the small cohort sizes, the varying assessment time points, the frequent absence of serial measurements, and selection bias toward those who survived and completed follow-up. Furthermore, Mattke et al. (34) conducted a single, long-term cross-sectional assessment; therefore, their study does not allow for the individual reconstruction of the trajectory following discharge. Consequently, the available evidence describes favourable changes during follow-up but does not allow us to assert that recovery is uniform or that it necessarily occurs during a specific period.

Rehabilitation could be part of this process, although its specific role remains understudied. Sirignano et al. (19) documented functional improvement in 84.6% of patients following their stay in a pediatric rehabilitation unit. Since this was a cohort study without a comparison group, this result does not allow us to attribute the improvement to the rehabilitation intervention. Nevertheless, it shows that the FSS can detect changes during recovery and highlights the need to prospectively evaluate access to, intensity of, and duration of rehabilitation during and after ECMO.

A return of the FSS to values close to 6 should not be equated with a full recovery either. Turner et al. (33) found that a more severely impaired FSS was associated with lower Vineland-3 scores at discharge (R² = 0.12) and during follow-up (R² = 0.24); furthermore, some patients with an overall FSS close to normal exhibited neurobehavioral deficits detected by the Vineland-3. Mattke et al. (34) supplemented the assessment with the PedsQL, although they did not formally analyse the association between the two instruments. Other studies linked functional outcomes to neurological lesions, ischemia, or seizures documented by neuroimaging or electroencephalography. These results provide evidence of clinical relevance but do not formally validate the FSS in this population.

The FSS offers practical advantages for describing functional dependence across six domains and can be applied using readily available clinical information. However, it has inherent limitations for the follow-up of ECMO survivors. Its normal functioning threshold is centred on a minimum score of 6, which may result in a floor effect and reduce its ability to distinguish between children with good functional independence. In clinical terms, this amounts to a “ceiling” on normal functioning: patients with differences in cognition, adaptive behaviour, school performance, or social participation may receive the same overall score. The scale also provides a limited assessment of cognition and subtle neurodevelopment, does not directly measure quality of life, and is not specific to a particular disease or form of life support. Therefore, while it is useful for describing overall dependency, it does not replace assessments of neurodevelopment, adaptive behaviour, quality of life, or social participation.

Determining baseline status is another limitation, particularly in neonates who had not reached an observable functional state outside the hospital setting before ECMO. In these patients, a baseline FSS score of 6 reflects a convention rather than an empirical measure of prior functioning. The studies used different thresholds, two, six, or ten days of life to apply this assignment, while others did not describe the procedure. In the post-Norwood cohorts, all participants were conventionally assigned a baseline score of 6. Because new morbidity is defined as the difference between the baseline and discharge values, decisions about this assignment directly influence the estimated frequency of deterioration. Standardising the neonatal baseline FSS is therefore a specific methodological priority.

Retrospective score reconstruction was the predominant procedure, but few studies reported the number and training of the raters, whether the assignment was performed independently, or how discrepancies were resolved. Sirignano et al. (19) were the only ones to formally quantify interobserver agreement and reported high values for baseline (*κ*=0.877) and discharge (*κ*=0.915). The CPCCRN/BATE analyses utilised trained coordinators, direct observation, discussion with the treating team, and review of the medical record McCabe et al. (32) conducted prospective, real-time assessments; Mattke et al. (34) used interviews with caregivers; and McGetrick et al. (24) relied on scores assigned during clinical practice. These procedures show that the FSS can be obtained from various sources, but they do not allow us to assume that the scores are interchangeable. The reproducibility of retrospective measurement was evaluated in only one study and remains insufficiently characterised.

Clinical heterogeneity prevents the identification of clearly distinct subpopulations. The evidence encompassed a continuum ranging from newborns with congenital heart disease, univentricular physiology, diaphragmatic hernia, or persistent pulmonary hypertension to older children with myocarditis, sepsis, acute respiratory failure, or ECPR. These populations differ in observable baseline function, neurological risks, the indication for support, and the relevance of the FSS domains. It was also not possible to establish functional differences between veno-arterial and veno-venous ECMO. Although the veno-arterial modality predominated and some studies included only one modality, most did not present FSS results stratified by support type. Beshish et al. (36). Despite including a nearly balanced distribution between VA and VV, they reported functional outcomes in an aggregated manner. Consequently, the review does not allow us to determine whether differences in function are related to the modality or to the diagnoses and indications that lead to the selection of each modality.

The lack of follow-up contrasts with the current emphasis on assessing survival beyond discharge. ELSO provides specific recommendations for follow-up after neonatal and pediatric ECMO and promotes longitudinal assessment of function, neurodevelopment, and quality of life (37). However, only eight of the 25 publications included any measurements after discharge or discontinuation of support; only two evaluated outcomes after five years, and only one follow-up cohort used a prospective design. Furthermore, the two publications with follow-up exceeding five years included 61 survivors in their original cohorts and functionally assessed even fewer. This gap limits the development of longitudinal benchmarks and makes it difficult to determine whether normal FSS is maintained, improves, or coexists with deficits that the scale does not detect.

Based on these findings, we can define specific priorities. Future studies should use serial measurements at baseline, discharge, three or six months, one year, and subsequent follow-up; establish a uniform standard for baseline neonatal function; report the source of the score, the training and number of evaluators, and interobserver agreement; and present results stratified by age, diagnosis, indication, and ECMO modality. It is also necessary to formally evaluate the validity, reliability, and responsiveness of the FSS in this population and to determine what magnitude of change represents a clinically meaningful difference. The ≥ 3-point threshold used to define new morbidity comes from the general pediatric intensive care literature and has not been validated specifically in ECMO survivors. The FSS should therefore be complemented with measures of neurodevelopment, adaptive behavior, quality of life, and social participation, particularly during long-term follow-up. Qualitative and mixed-methods studies are also needed to explore patients' and families' experiences, changes in family dynamics, caregiving and rehabilitation needs, and the influence of the social, educational, and community environment on recovery after ECMO. Finally, comparative cohorts of critically ill patients not receiving ECMO are required to distinguish, at least partially, the contribution of extracorporeal support from that of critical illness itself.

This review has limitations. The search may have excluded unindexed publications, and data extraction depended on the level of detail reported by the authors, which was particularly limited in the three conference proceedings. In keeping with the purpose of a scoping review, no formal assessment of risk of bias was conducted; therefore, the results allow for mapping and characterising the evidence but do not enable the formulation of clinical recommendations. The unadjusted total of the samples was approximately 4,900 patients, visits, encounters, or surveys; however, this figure does not represent unique individuals: four publications came from CPCCRN/BATE, two used the same post-Norwood cohort, another two analyzed the same VA-ECLS cohort, and the candidate studies were derived from the same parent study. The volume of independent evidence is therefore smaller than the aggregate number of records suggests. The fact that 21 of the 25 publications are from the United States also limits the representation of other healthcare systems and rehabilitation and follow-up contexts.

In summary, the reviewed evidence shows that a significant proportion of neonatal and pediatric ECMO survivors experience functional impairment at discharge, generally mild or moderate, but associated with clinically relevant needs for feeding, respiratory support, medical devices, and rehabilitation. Follow-up studies suggest that some of these limitations may diminish over the subsequent months or years; however, recovery was not uniform, and an overall score close to normal could coexist with neurobehavioral, adaptive, or quality-of-life impairments. Significant gaps persist due to limited longitudinal follow-up, non-standardised assessment of baseline neonatal function, predominantly retrospective reconstruction of scores, limited reporting of domains, and the absence of results stratified by age, diagnosis, indication, and ECMO modality. In light of these findings, prospective, multicenter studies with serial measurements and standardised procedures are needed, combining the FSS with assessments of neurodevelopment, adaptive behaviour, quality of life, and social participation. This approach should be complemented by qualitative studies that incorporate the experiences of patients and their families, care and rehabilitation needs, and the influence of the social, educational, and community environment. Such an approach could provide a more comprehensive understanding of the recovery trajectories and needs of ECMO survivors and their families, extending beyond the assessment of functional status at discharge.

## 5 Conclusions

The available evidence suggests that the FSS is a useful tool for assessing functional status in pediatric patients undergoing ECMO, particularly in the hospital setting. However, its current application is primarily limited to describing functional changes, with little standardization in its use and limited exploration of its prognostic value and clinical utility. Future studies are needed to strengthen the evaluation of its metric properties in this population, standardize its application, and explore its potential to guide early interventions and rehabilitation strategies, with the aim of improving long-term functional outcomes in this population.

## Data Availability

The original contributions presented in the study are included in the article/Supplementary Material, further inquiries can be directed to the corresponding author.
