# Correction: Functional assessment in children undergoing extracorporeal membrane oxygenation using the functional status scale: a scoping review

**DOI:** 10.3389/fped.2026.2016685

**Published:** 2026-10-28

**Authors:** Jhon Jairo Botello Jaimes, Luisa Fernanda Cornejo Castaño, Luis Fernando Sánchez Estrada, Angie Katherine Turriago Castañeda

**Affiliations:** 1 Facultad de Ciencias de la Salud, Corporación Universitaria Remington, Medellin, Colombia; 2 Facultad de Ciencias de la Salud, Universidad ICESI, Cali, Colombia

**Keywords:** adolescent, children, extracorporeal membrane oxygenation, functional status scale, infant, neonate, pediatric intensive care

Author Angie Katherine Turriago Castañeda was erroneously included as corresponding author. The correct corresponding author is Jhon Jairo Botello Jaimes.

The original version of this article has been updated.

