# Supplementary material for: Functional assessment in children undergoing extracorporeal membrane oxygenation using the functional status scale: a scoping review
Source: Front Pediatr. 2026 Sep 23;14:1910668. doi: 10.3389/fped.2026.1910668 (PMC13642717; doi:10.3389/fped.2026.1910668)
Supplement: Supplementary file 1 [file Table1.docx]

Supplementary Material

# 1 Supplementary Table

**Supplementary Table 1. Search strategy**

| **Database** | **Search equation** | **Results** |
| --- | --- | --- |
| **PubMed** | (("functional status"[MeSH Terms] OR "functional status scale"[Title/Abstract]) AND ("Extracorporeal life support"[Title/Abstract] OR "extracorporeal membrane oxygenation"[MeSH Terms]) AND ("child"[MeSH Terms] OR "pediatrics"[MeSH Terms] OR "infant"[MeSH Terms] OR "infant, newborn"[MeSH Terms])) | 19 |
| **Ovid Emcare** | (exp Functional Status/ OR "functional status scale".ti,ab.) AND (exp Extracorporeal Membrane Oxygenation/ OR "extracorporeal life support".ti,ab.) AND (exp Child/ OR exp Pediatrics/ OR exp Infant/ OR exp Infant, Newborn/) | 71 |
| **Scopus** | TITLE-ABS-KEY ( ( "functional status scale" OR FSS ) AND (ECMO OR "extracorporeal membrane oxygenation" ) AND (pediatric* OR paediatric* OR child* OR infant* OR neonat* )) | 28 |
